# Pharmacological intervention for ambulatory surgery: A protocol for systematic review and network meta-analysis

**DOI:** 10.1097/MD.0000000000021580

**Published:** 2020-08-07

**Authors:** Geun Joo Choi, Hee-Kyeong Seong, Hyun Kang

**Affiliations:** Department of Anesthesiology and Pain Medicine, Chung-Ang University College of Medicine, Seoul, Republic of Korea.

**Keywords:** ambulatory surgery, analgesics, network meta-analysis, pain, postoperative, systematic review

## Abstract

**Background:**

We aim to perform a network meta-analysis (NMA) to quantify and rank-order the efficacy and safety of analgesic medications for ambulatory surgery.

**Methods:**

We will search MEDLINE, EMBASE, the Cochrane Central Register of Controlled Trials, and Google Scholar databases to identify all randomized controlled trials (RCTs) of analgesics, beginning from their inception to February 2020. The primary endpoints will be pain score measured using a visual analog scale (VAS) or a numerical rating scale (NRS) at 3 different time points: Phase I recovery, phase II recovery, and recovery at home. Adverse events, including nausea, vomiting, headache, dizziness, arrhythmia, and respiratory depression, will be also assessed.

We will conduct NMA and use surface under the cumulative ranking curve (SUCRA) values and rankograms to present the hierarchy of analgesic medication. A comparison-adjusted funnel plot will be used to assess the presence of small study effects. The quality of the included studies will be assessed using the risk of bias tool 2.0. All statistical analyses will be performed using Stata SE version 15.0.

**Results:**

The results of this systematic review and NMA will be published in a peer-reviewed journal.

**Conclusion:**

This systematic review and NMA will provide comprehensive and convincing evidence regarding analgesic medication for pain after ambulatory surgery.

**Trial registration number:**

CRD42018100000.

## Abstract 2

Supplemental Digital Content is available in the text

## 1 Introduction

Adequate postoperative pain control is an essential part of ambulatory surgery, and inadequate analgesia after ambulatory surgery delays discharge and results in extended convalescence in the recovery room. Poor pain relief is associated with undesirable conditions after discharge, including hospital readmission. Insufficient analgesia after discharge can cause limitation of early mobility and a subsequent delay in the return to normal function. Postoperative pain control is crucial both before and after discharge, particularly in ambulatory settings.

Traditionally, ambulatory surgery is performed in patients classified as American Society of Anesthesiology (ASA) I or II. Recently, patients with an advanced ASA classification undergo surgery and receive anesthesia through an ambulatory approach.^[1]^ The surgical volume is progressively increased in a broad range of patients, which makes adequately effective and safe analgesia after ambulatory surgery even more important.

A number of medications for pain management have been used for adequate analgesia after ambulatory surgery, and many researchers have reported the efficacy and safety regarding analgesic medications in ambulatory settings. However, the relative efficacy and safety of the majority of these analgesics remain unknown. Therefore, we planned to conduct a systematic review and network meta-analysis (NMA) of published studies to comprehensively quantify and rank-order the efficacy and safety of analgesic medications for pain management after ambulatory surgery.

## 2 Methods

### 2.1 Protocol design and registration

Our systematic review and meta-analysis protocol was developed following the Preferred Reporting Items for Systematic Review and Meta-Analysis Protocols (PRISMA-P) statement.^[2]^

The protocol for this systematic review and NMA is registered with the International Registration of Prospective Systematic reviews (PROSPERO network) and assigned the registration number CRD42018100000, the record of which can be accessed on their website (https://www.crd.york.ac.uk/prospero/display_record.php?ID=CRD42018100000).

The present systematic review and meta-analysis will be conducted according to the protocol recommended by the Cochrane Collaboration^[3]^ and will be presented following the PRISMA guidelines for reporting NMA.^[4]^

### 2.2 Inclusion and exclusion criteria

#### 2.2.1 Types of studies

Peer-reviewed randomized clinical studies will be eligible for inclusion. No language or date restrictions will be applied. Review articles, case reports, case series, letters to the editor, commentaries, proceedings, laboratory science studies, and any other non-relevant studies will be excluded from analysis.

#### 2.2.2 Population

The inclusion criteria for the study populations will be as follows:

1. Patients undergoing elective ambulatory surgery under general anesthesia or sedation and
2. patients who received analgesics for pain control.

#### 2.2.3 Intervention and comparison

Examination of interventions and comparisons will include all types of analgesics, including nonsteroidal anti-inflammatory drugs (NSAIDs), opioids, cyclooxygenase-2 (COX-2) inhibitors, and acetaminophen.

#### 2.2.4 Outcome

1. Effectiveness

The primary endpoint will be the pain score measured using a visual analog scale (VAS) or a numerical rating scale (NRS) at a post anesthetic care unit (PACU), in phase 2, and at home.

1. Safety

Adverse events, including nausea, vomiting, headache, dizziness, arrhythmia, and respiratory depression, will also be assessed.

#### 2.2.5 Information sources

##### 2.2.5.1 Electronic search

A search will be performed in MEDLINE, EMBASE, the Cochrane Central Register of Controlled Trials (CENTRAL), and Google Scholar using search terms related to ambulatory surgery and analgesics. Search terms to be used for MEDLINE and EMBASE are presented in the Supplemental Digital Content (Appendix). Reference lists will be imported into Endnote software (Thompson Reuters, CA, USA) and duplicate articles will be removed. Additional relevant articles will be identified by scanning the reference lists of articles found from the original search.

##### 2.2.5.2 Study selection

The titles and abstracts identified through the search strategy described above will be scanned independently by 2 authors. To minimize data duplication due to multiple reporting, papers from the same author will be compared. In reports that are determined to be eligible based on the title or abstract, the full paper will be retrieved. Potentially relevant studies chosen by at least one author will be retrieved and evaluated as full-text versions. Articles meeting the inclusion criteria will be assessed separately by 2 authors, and any discrepancies will be resolved through discussion. In cases where an agreement cannot be reached, the dispute will be resolved with the help of a third investigator. A flow diagram for the search and selection process will be developed following the PRISMA guidelines.

#### 2.2.6 Data extraction

Using a standardized extraction form, the following data will be extracted independently by 2 authors: study name (along with the name of the first author and year of publication); country where the study was conducted; name of journal; study design; type of surgery; type of analgesic; dose of analgesic; number of subjects; pain measured as VAS or NRS scores at the PACU, in phase 2, and at home; use of additional analgesics; and incidence of adverse events including nausea, vomiting, headache, dizziness, arrhythmia, and respiratory depression.

If information is missing, an attempt will be made to contact the study authors to obtain the relevant information. If some data are presented as figures rather than numbers, the open source software Plot Digitizer (version 2.6.8; http://plotdigitizer.sourceforge.net) will be used to extract the numbers. When unsuccessful, missing information will be calculated if possible from the relevant data within the study. The reference list will be divided in 2, and 2 authors will complete data extraction for each half of the list. Data extraction forms will then be cross-checked to verify the accuracy and consistency of the extracted data.

The degree of agreement between the 2 independent data extractors (Seong HK and Choi GJ) will be computed using kappa statistics to measure the difference between the observed and expected agreements between Seong HK and Choi GJ; namely, whether they were at random or by chance only. Kappa values will be interpreted as follows:

1. less than 0: less than chance agreement;
2. 0.01–0.20: slight agreement;
3. 0.21–0.40: fair agreement;
4. 0.41–0.60: moderate agreement;
5. 0.61–0.80: substantial agreement; and
6. 0.8–0.99: almost perfect agreement.^[5]^

#### 2.2.7 Study quality assessment

The quality of the studies will be independently assessed by 2 of the papers authors (Choi GJ and Kang H), using the Revised Cochrane risk of bias tool for randomized trials (RoB 2.0).^[6]^ The risk of bias (ROB) will be evaluated by considering the following 5 potential sources of bias:

1. bias arising from the randomization process;
2. bias due to deviations from intended interventions;
3. bias due to missing outcome data;
4. bias in measurement of the outcome; and
5. bias in selection of the reported result.

Then, we will evaluate the overall ROB judgment according to these domain-level judgments. The methodology for each domain will be graded as “low ROB,” “some concerns,” or “high ROB”.^[6]^

### 2.3 Statistical analysis

Ad-hoc tables will be designed to summarize data from the included studies and show their key characteristics and any important questions related to the aim of this systematic review and NMA. After data are extracted, reviewers will determine whether an NMA is possible; this will involve evaluating the transitivity assumption by examining the comparability of patient eligibility criteria, pertinent patient demographics, type of surgery, and ROB (all vs removing high ROB for bias arising from the randomization process, and bias in measurement of the outcome) as potential treatment-effect modifiers across comparisons.^[7]^ The methodological differences between studies that could influence outcome measurements and any concerns related to the transitivity assumption or methodological heterogeneity will be noted.

We will evaluate the treatment nodes from a connected network of evidence, and if the treatment node is connected, we will perform an NMA. A multiple treatment comparison NMA is a generalization of meta-analysis methods that includes both direct RCT comparisons and indirect comparisons of treatments. An NMA based on a frequentist framework will be performed with NMA graphical tools by Chaimani et al.^[8]^ Given the clinical and methodological heterogeneity of the populations and methods among the included trials in NMAs, we will use the random-effects model in our primary analyses.

A network plot linking all the included analgesics will be formed to indicate the type of analgesics, the number of patients receiving different analgesics, and the pairwise comparisons. In the network plot, nodes will show the analgesic being compared and the edges will show the available direct comparison between analgesics. Each drug, as well as each combination of drugs, will be treated as a node in this network. Nodes and edges will be weighted according to the number of patients and inverse of standard error (SE), respectively.

We will examine the consistency of the total network through both global and local tests of inconsistency. We will evaluate the global consistency assumption using the design-by-treatment interaction model,^[9]^ and we will also evaluate each closed loop in the network to evaluate local inconsistency between direct and indirect effect estimates for the same comparison. In each loop, we will estimate the inconsistency factor (IF) as the absolute difference (with 95% CI and a z-test) between direct and indirect estimates for each paired comparison in the loop. IF is the logarithm of the ratio of 2 odds ratios (RoR) from direct and indirect evidence in the loop; RoR values close to 1 indicate that the 2 sources are in agreement.

We will also show the relative treatment effects between all active medications in ranked forest plots. Mean summary effects with confidence intervals (CIs) will be presented together with their predictive intervals (PrIs) to facilitate interpretation of the results in the light of the magnitude of heterogeneity. PrIs provide an interval, which is expected to encompass the estimate of a future study. We will not adjust for multiple comparisons in successive NMAs as we are not interested in establishing the superiority or inferiority of particular comparisons.

A rankogram and cumulative ranking curve will be drawn for each analgesic. A rankogram plots the probabilities for treatments to assume any of the possible ranks, i.e., the probability that a given treatment ranks first, second, third, and so on, among all treatments evaluated in the NMA. We will use the surface under the cumulative ranking curve (SUCRA) values to present the hierarchy of interventions. SUCRA is a relative ranking measure that accounts for the uncertainty in treatment order, i.e., it accounts both for the location and the variance of all relative treatment effects.^[10]^ A higher SUCRA value is regarded as a better result for individual interventions, and while ranking treatments, the closer a percentage value is to 100%, the higher is the treatment ranking, relative to all other treatments.

We will test small study effects and publication bias using the comparison-adjusted funnel plot.^[11]^

An NMA will first be performed based on data derived purely from RCTs for each drug or combination of drugs, and data will subsequently be categorized into types of drugs (opioid agonist, opioid partial agonist, NSAIDs, COX-2 inhibitor, and combinations of each); the analyses are run a second time such that the effects of these data are apparent to the readers.

If clinical and methodological heterogeneity between the study arms are found to be substantial, we will present pairwise meta-analysis. If the transitivity assumption cannot be adequately met, a descriptive summary of the study findings will be presented. If inconsistency for the entire network or local inconsistency is suspected, we will conduct sensitivity analyses to evaluate the reason for inconsistency and the influence of individual studies on the overall effect estimate by excluding one study at a time from the analysis. All statistical analyses will be performed using Stata SE version 15.0 (StataCorp, College Station, TX).

### 2.4 Evidence synthesis

The overall quality of evidence for each outcome assessed will be rated using the guidelines developed by the Grading of Recommendations Assessment, Development, and Evaluation working group, designed for rating the quality of effect estimates derived from a NMA. These guidelines use sequential assessment of the evidence quality followed by an assessment of the risk-benefit balance and a subsequent judgment on the strength of the recommendations.^[12]^ We will use a 4-step process:

1. present direct and indirect treatment estimates (mean differences, standardized mean differences, or RRs with 95% CIs);
2. rate the quality of direct and indirect treatment estimates;
3. present NMA estimates (pool of direct and indirect estimates, mean differences, standardized mean differences or RRs with 95% CIs); and
4. rate the quality of NMA estimates.

### 2.5 Ethics and dissemination

#### 2.5.1 Ethical issues

This systematic review does not require an ethics approval or informed consent because there will be no direct contact with individual patients. Only previously published data will be included in the review.

#### 2.5.2 Publication plan

This systematic review will be published in a peer-reviewed journal and will be disseminated electronically and in print.

## 3 Discussion

Ambulatory surgery has shown a positive trend in health care with the improvement of surgical equipment, volatile anesthetics, regional anesthetic technique, and medications such as analgesics and antiemetics.^[13]^ The reduction in hospital stay lowers the risk of community infection, provides patients with the convenience of undergoing surgeries without hospital admission, and decreases the number of days missed from work.^[14]^

In a study on postoperative pain, 78% of patients undergoing ambulatory surgery experienced pain in the recovery phase, which indicates the crucial role of pain control after ambulatory surgery.

In the outpatient setting, the recovery process is divided into 3 distinct phases: early recovery, from the discontinuation of anesthetics to the recovery of protective reflexes and motor function; intermediate recovery, when the patient fulfills the criteria for discharge; and late recovery, when patients return home expecting to return to their preoperative physiological state.^[15]^ Thus, we plan to evaluate outcomes, such as pain scores and complications, according to the early, intermediate, and late recovery phases. By assessing the outcomes from the immediate period after surgery to the period after discharge, we will be able to evaluate and compare the efficacy and safety of analgesics for ambulatory surgery across the board.

The purpose of this study is to suggest a clinically useful ranking of pharmacological interventions for pain control following ambulatory surgery and to provide evidence for physicians that will guide them toward clinical decisions in terms of enhancing the efficacy and safety of analgesic medication.

## Author contributions

**Data curation:** Choi GJ, Seong HK.

**Funding acquisition:** Kang H.

**Investigation:** Choi GJ, Seong HK.

**Methodology:** Choi GJ, Kang H.

**Project administration:** Kang H.

**Writing – original draft:** Seong HK, Choi GJ.

**Writing – review & editing:** Kang H.

## Supplementary Material

Supplemental Digital Content
