# Supplementary material for: Pharmacological intervention for ambulatory surgery: A protocol for systematic review and network meta-analysis
Source: Medicine (Baltimore). 2020 Aug 7;99(32):e21580. doi: 10.1097/MD.0000000000021580 (PMC7593075; doi:10.1097/MD.0000000000021580)
Supplement: Supplemental Digital Content [file medi-99-e21580-s001.docx]

**Appendix**

**Search terms for MEDLINE**

1. randomized controlled trial.pt
2. randomized controlled trial$.mp
3. controlled clinical trial.pt
4. controlled clinical trial$.mp
5. random allocation.mp
6. exp double-blind method/
7. double-blind.mp
8. exp single-blind method/
9. single-blind.mp
10. or/1-9
11. clinical trial.pt
12. clinical trial$.mp
13. exp clinical trial/
14. (clin$ adj25 trial$).mp
15. ((singl$ or doubl$ or tripl$ or trebl$) adj25 (blind$ or mask$)).mp
16. random$.mp
17. exp research design/
18. research design.mp
19. or/11-18
20. 10 or 19
21. case report.tw
22. letter.pt
23. historical article.pt
24. review.pt
25. or/21-24
26. 20 not 25
27. exp outpatients/
28. outpatient.mp
29. ambulatory.mp
30. one day.mp
31. one-day.mp
32. day case.mp
33. day-case.mp
34. same-day.mp
35. same day.mp
36. or/27-35
37. procedure.mp
38. intervention.mp
39. anesthesia.mp
40. anaesthesia.mp
41. surgery.mp
42. operation.mp
43. or/37-42
44. exp Ambulatory Surgical Procedures/
45. day surgery.mp
46. or/43-45
47. 26 and 46
48. exp acute pain/
49. exp analgesics/
50. exp analgesics, opioid/
51. fentanyl.mp
52. sufentanil.mp
53. alfentanil.mp
54. remifentanil.mp
55. oxycodone.mp
56. morphine.mp
57. nalbuphine.mp
58. pethidine.mp
59. codeine.mp
60. butorphannol.mp
61. dihydrocodeine.mp
62. buprenorphine.mp
63. dezocin.mp
64. exp Anti-Inflammatory Agents, Non-Steroidal/
65. ketorolac.mp
66. naproxen.mp
67. ibuprofen.mp
68. dextropropoxyphene.mp
69. ketoprofen.mp
70. fenoprofen.mp
71. diclofenac.mp
72. tenoxicam.mp
73. meloxicam.mp
74. indomethacin.mp
75. exp acetaminophen/
76. paracetamol.mp
77. metamizole.mp
78. exp Cyclooxygenase 2 Inhibitors/
79. parecoxib.mp
80. rofecoxib.mp
81. celecoxib.mp
82. etoricoxib.mp
83. aspirin.mp
84. acetylsalicylate.mp
85. tramadol.mp
86. gabapentin.mp
87. exp pregabalin/
88. nefopam.mp
89. or/48-88
90. exp dexamethasone/
91. exp betamethasone/
92. prednisone.mp
93. esmolol.mp
94. diphenhydramine.mp
95. lidocaine.mp
96. magnesium.mp
97. dexmedetomidine.mp
98. midazolam.mp
99. exp ketamine/
100. diazepam.mp
101. buscopan.mp
102. or/90-101
103. exp pain/
104. 102 and 103

**Search terms for EMBASE**

1. randomi?ed controlled trial$.mp.
2. 'controlled clinical trial (topic)'/exp
3. controlled AND clinical AND trials
4. controlled clinical trial$.mp.
5. 'randomization'/exp
6. 'random allocation'/exp
7. random allocation.mp.
8. double-blind.mp.
9. single-blind.mp.
10. #1 OR #2 OR #3 OR #4 OR #5 OR #6 OR #7 OR #8 OR #9
11. 'clinical trial (topic)'/exp
12. clinical AND trial$.mp.
13. random$.mp.
14. rct
15. #11 OR #12 OR #13 OR #14
16. #10 OR #15
17. 'case study'/exp
18. 'case report'/exp
19. 'abstract report'/exp
20. 'letter'/exp
21. #17 OR #18 OR #19 OR #20
22. #16 NOT #21
23. 'outpatient'/exp
24. outpatient
25. ambulatory
26. ‘one day’
27. One-day
28. ‘day case’
29. day-case
30. same-day
31. ‘same day’
32. #23 OR #24 OR #25 OR #26 OR #27 OR #28 OR #29 OR #30 OR #31
33. procedure
34. intervention
35. anesthesia
36. anaesthesia
37. surgery
38. operation
39. #33 OR #34 OR #35 OR #36 OR #37 OR #38
40. #32 AND #39
41. 'ambulatory surgery'/exp
42. 'ambulatory surgery'/exp
43. #22 AND #42
44. 'analgesic agent'/exp
45. ‘opiate’/exp
46. ‘narcotic analgesic agent’/exp
47. 'morphine derivative'/exp
48. 'fentanyl derivative'/exp
49. Fentanyl
50. morphine
51. Alfentanil
52. Sufentanil
53. Remifentanil
54. Buprenorphine
55. Meperidine
56. Pethidine
57. Nalbuphine
58. pentazocine
59. butorphanol
60. codeine
61. oxycodone
62. hydromorphone
63. oxynorm
64. dezocine
65. #45 OR #46 OR #47 OR #48 OR #49 OR #50 OR #51 OR #52 OR #53 OR #54 OR #55 OR #56 OR #57 OR #58 OR #59 OR #60 OR #61 OR #62 OR #63 OR #64
66. 'nonsteroid antiinflammatory agent'/exp
67. ketorolac
68. naproxen
69. ibuprofen
70. dextropropoxyphene
71. ketoprofen
72. fenoprofen
73. diclofenac
74. tenoxicam
75. meloxicam
76. indomethacin
77. nsaid
78. 'paracetamol'/exp
79. acetaminophen
80. metamizole
81. #66 OR #67 OR #68 OR #69 OR #70 OR #71 OR #72 OR #73 OR #74 OR #75 OR #76 OR #77 OR #78 OR #79 OR #80
82. 'cyclooxygenase 2 inhibitor'/exp
83. 'cox 2'
84. parecoxib
85. rofecoxib
86. celecoxib
87. etoricoxib
88. #82 OR #83 OR #84 OR #85 OR #86 OR #87
89. aspirin
90. acetylsalicylate
91. tramadol
92. 'gabapentin'/exp
93. 'pregabalin'
94. nefopam
95. Dexamethasone
96. betamethasone
97. ketamine
98. diphenhydramine
99. lidocaine
100. magnesium
101. dexmedetomidine
102. midazolam
103. #89 OR #90 OR #91 OR #92 OR #93 OR #94 OR #95 OR #96 OR #97 OR #98 OR #99 OR #100 OR #101 OR #102
104. #44 OR #65 OR #81 OR #88 OR #103
105. #43 AND #104
